# Outbreak of SARS-CoV-2 Omicron Infection in a Centralized Quarantine Location in Hangzhou, China

**DOI:** 10.1001/jamanetworkopen.2022.47219

**Published:** 2022-12-16

**Authors:** Hexiang Jia, Yongtao Zheng, Yuxin Jia, Chenyang Jin, Yingjian Wang, Jiaqin Zhuang, Yang Ge

**Affiliations:** 1 Department of Infectious Disease, Xiaoshan Center for Disease Control and Prevention, Hangzhou, China; 2 Department of General Thoracic Surgery, The First Affiliated Hospital, College of Medicine, Zhejiang University, Hangzhou, China; 3 Department of Public Health, Community Healthcare Center of Guali Town, Hangzhou, China; 4 School of Health Professions, University of Southern Mississippi, Hattiesburg

## Abstract

This cohort study assesses whether transmission of COVID-19 occurred among individuals staying on different floors at a hotel used as a centralized quarantine location in Hangzhou, China.

## Introduction

Infection with SARS-CoV-2 may be transmitted between people more than 2 m apart.^1,2^ In 2021, a more transmissible variant, Omicron, was identified,^3^ leading to challenges in centralized quarantine. Hotels were commonly used as centralized quarantine locations to reduce community transmission. However, intra-hotel outbreaks were reported.^4^ Transmission may have been attributable to the leaking of contaminated aerosol from a patient’s room to other rooms on the same floor.^5^ In this study, we investigated an outbreak related to rooms on different floors.

## Methods

This outbreak investigation followed the STROBE reporting guideline. The outbreak was observed in a centralized quarantine location (a hotel) in Hangzhou, China. COVID-19 was confirmed by reverse transcriptase–polymerase chain reaction (RT-PCR). All individuals tested negative within 48 hours before arrival and received a test every day. They were not allowed to leave their rooms and were required to wear a surgical mask while opening the room door for food delivery and garbage disposal. This study was approved by the Xiaoshan Center for Disease Control and Prevention, Hangzhou Ethics Committee. Written informed consent was obtained from all participants.

The hotel requisitioned for COVID-19 quarantine has a courtyard—a space surrounded by walls and windows of rooms on different floors. The courtyard has a length of around 22.5 m, width of around 0.96 m, and height of around 10.0 m (Figure, A and B). It facilitates lighting and allows airflow. Therefore, windows of adjacent rooms are nearby (<1 m). Without a central air conditioner, each room has an independent wall-mounted air conditioner.

**Figure.**
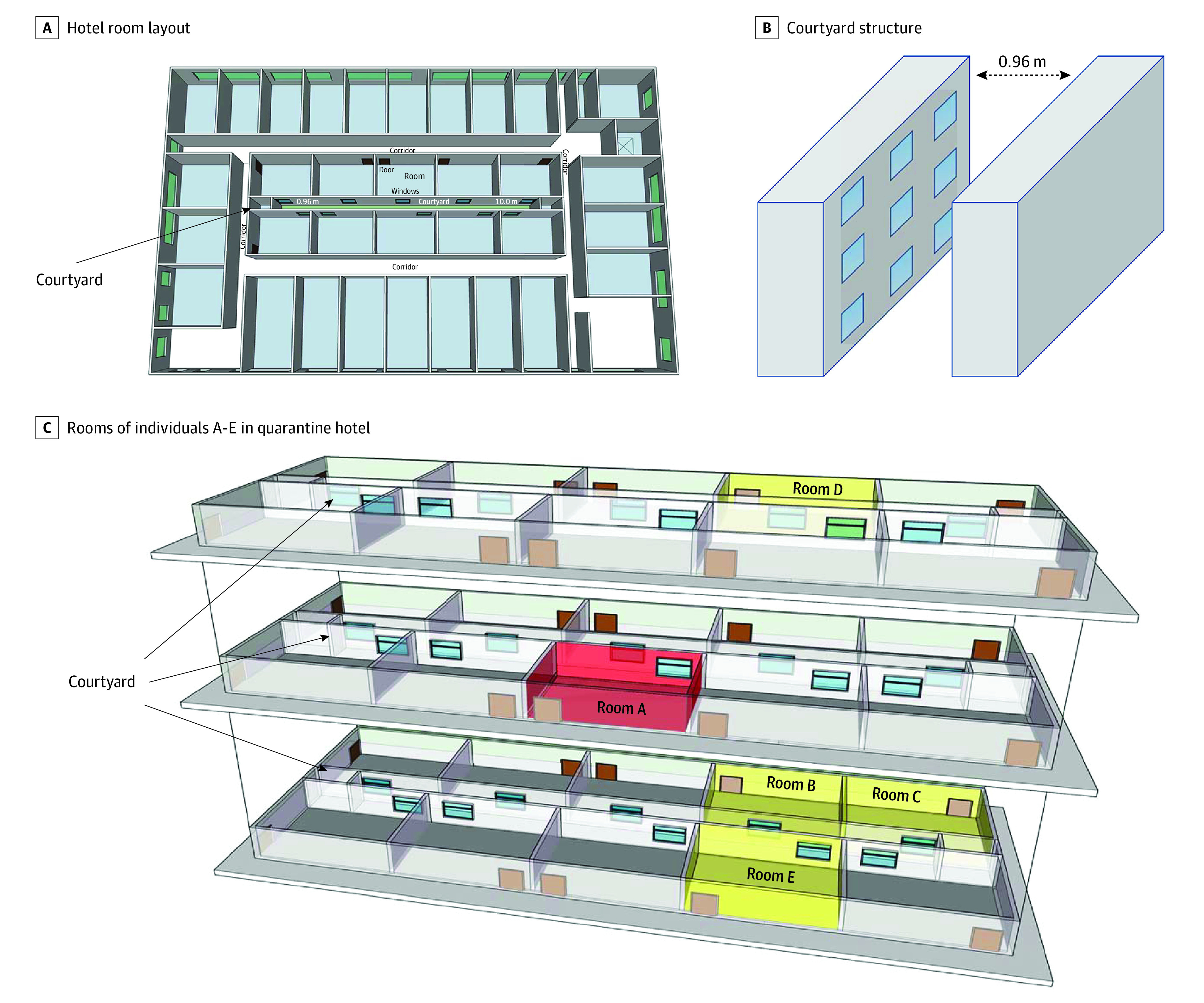
Hotel Structure and Case Distribution Individuals A, B, C, D, and E lived in rooms A, B, C, D, and E, respectively.

## Results

Five individuals (all male; mean [SD] age, 37 [6] years) arrived at the quarantine hotel successively and were located in different rooms on different floors around the courtyard (Table and Figure, C). Individual A was transferred (room A) on April 13, 2022. He smoked by the window during quarantine. He tested positive for COVID-19 by RT-PCR and was diagnosed as an asymptomatic carrier on April 16. Individual B (room B) and individual C (room C) were transferred on April 11 and April 13, respectively, and tested positive on April 21. Individuals D and E were transferred on April 14 (rooms D and E, respectively) and tested positive on April 21 (7 days later). Individuals B, C, D, and E had different travel histories than individual A.

**Table.** Basic Information of Individuals in the Quarantine Hotel

Individual/sex/age range	Location	Date entered quarantine	Date of positive test result	Symptoms at hospital admission	SARS-CoV-2 gene sequencing result	Other clear epidemiological source of SARS-CoV-2 infection outside the hotel
A/male/40s	Room A; third floor	April 13, 2022	April 16, 2022	None	Omicron variant (BA.2.2 lineage)	NA
B/male/30s	Room B; second floor	April 11, 2022	April 21, 2022	None	Omicron variant (BA.2.2 lineage)	None
C/male/40s	Room C; second floor	April 13, 2022	April 21, 2022	None	Omicron variant (BA.2.2 lineage)	None
D/male/30s	Room D; fourth floor	April 14, 2022	April 21, 2022	None	Omicron variant (BA.2.2 lineage)	None
E/male/30s	Room E; second floor	April 14, 2022	April 21, 2022	None	None	None

Abbreviation: NA, not applicable.

A total of 23 COVID-19 cases (including A, B, C, D, and E) were confirmed from April 14 to April 21. Viral gene sequencing showed that 15 individuals (including A, B, C, and D) were infected with the Omicron variant (BA.2.2 lineage). The genetic sequences of individuals A, B, and C were identical, and they were highly homologous to D, with only 1 nucleotide difference. Gene sequencing results of 11 individuals were inconsistent with those of individuals A, B, C, and D. The remaining 8 cases (including E) had no sequence data because the sequencing technology failed to deliver a result due to low SARS-CoV-2 load. Individuals B, C, D, and E tested positive by RT-PCR 7 days after arrival, which supported intra-hotel transmission given that the incubation period of the Omicron variant around 3 days.^6^ Windows of rooms B, C, D, and E were often open. Individuals B, C, and D reported that they smelled smoke during quarantine. Surveys of all individuals and records of security cameras showed no epidemiological evidence of other transmission exposures.

## Discussion

This outbreak of COVID-19 may have been attributable to transmission through a courtyard by activities such as smoking and opening windows. We call for attention on building structures for the selection of centralized quarantine locations. Hotels requisitioned for COVID-19 quarantine were not designed for quarantine, especially for airborne-transmissible infectious diseases. These centralized quarantine locations could be transmission hotspots. A study limitation is that 8 cases had no sequence data.

## Notes

Supplement.
Data Sharing Statement
