# Supplementary material for: Outbreak of SARS-CoV-2 Omicron Infection in a Centralized Quarantine Location in Hangzhou, China
Source: JAMA Netw Open. 2022 Dec 16;5(12):e2247219. doi: 10.1001/jamanetworkopen.2022.47219 (PMC9856702; doi:10.1001/jamanetworkopen.2022.47219)
Supplement: Supplement. — Data Sharing Statement [file jamanetwopen-e2247219-s001.pdf]

Data Sharing Statement

Jia. Outbreak of SARS-CoV-2 Omicron Infection in a Centralized Quarantine Location in Hangzhou, China. *JAMA Netw Open*. Published December 16, 2022. doi:10.1001/jamanetworkopen.2022.47219

Data
Data available: No
